# Bacurd1/Kctd13 and Bacurd2/Tnfaip1 are interacting partners to Rnd proteins which influence the long-term positioning and dendritic maturation of cerebral cortical neurons

**DOI:** 10.1186/s13064-016-0062-1

**Published:** 2016-03-11

**Authors:** Ivan Gladwyn-Ng, Lieven Huang, Linh Ngo, Shan Shan Li, Zhengdong Qu, Hannah Kate Vanyai, Hayley Daniella Cullen, John Michael Davis, Julian Ik-Tsen Heng

**Affiliations:** EMBL-Australia, The Australian Regenerative Medicine Institute, Monash University, Wellington Road, Clayton, VIC 3800 Australia; The Harry Perkins Institute of Medical Research, 6 Verdun St, Crawley, WA 6009 Australia; The Centre for Medical Research, The University of Western Australia, Crawley Avenue, Crawley, WA 6009 Australia

**Keywords:** Neuronal migration, Dendritic branching, Dendritic spines, Bacurd

## Abstract

**Background:**

The development of neural circuits within the embryonic cerebral cortex relies on the timely production of neurons, their positioning within the embryonic cerebral cortex as well as their terminal differentiation and dendritic spine connectivity. The RhoA GTPases Rnd2 and Rnd3 are important for neurogenesis and cell migration within the embryonic cortex (Nat Commun 4:1635, 2013), and we recently identified the **B**TB/POZ domain-containing **A**daptor for **Cu**l3-mediated **R**hoA **D**egradation family member Bacurd2 (also known as Tnfaip1) as an interacting partner to Rnd2 for the migration of embryonic mouse cortical neurons (Neural Dev 10:9, 2015).

**Findings:**

We have extended this work and report that Bacurd1/Kctd13 and Bacurd2/Tnfaip1 are interacting partners to Rnd2 and Rnd3 in vitro. Given that these genes are expressed during cortical development, we performed a series of *in utero* electroporation studies in mice and found that disruptions to *Bacurd1/Kctd13* or *Bacurd2/Tnfaip1* expression impair the long-term positioning of E14.5-born cortical neurons within the postnatal (P17) mouse cerebral cortex. We also find that forced expression of Bacurd1/Kctd13 and Bacurd2/Tnfaip1 alters the branching and dendritic spine properties of layer II/III projection neurons.

**Conclusions:**

We identify Bacurd1/Kctd13 and Bacurd2/Tnfaip1 as interacting partners to Rnd proteins which influence the development of cortical neurons. Their neurodevelopmental functions are likely to be relevant to human brain development and disease.

**Electronic supplementary material:**

The online version of this article (doi:10.1186/s13064-016-0062-1) contains supplementary material, which is available to authorized users.

## Introduction

During development, newborn neurons undergo directional cell migration to position themselves appropriately within the embryonic cerebral cortex before establishing their branching characteristics and finally establishing dendritic spine connections (reviewed in [1, 2]). Members of the Rnd family of RhoA GTPases such as Rnd2 and Rnd3 play critical roles in the production of neurons [3, 4] as well as their migration within the embryonic cortex [4–7]. On the other hand, Bacurd proteins comprise Bacurd1/Kctd13, Bacurd2/Tnfaip1 and Bacurd3/Kctd10, and the functions of these proteins are increasingly recognised to be relevant to the development of neurons as well as in synaptic signalling [8–10]. Indeed, elevations in the dosage of *BACURD1* and *BACURD2* are associated with human neurodevelopmental disorders [11–13], but the pathological consequences of *BACURD* overexpression in neurons remain poorly understood. We recently identified Bacurd2/Tnfaip1 as an interacting partner to Rnd2 which influences neuronal migration in a concentration-sensitive manner, and our studies provided the first insight into the potential roles for Bacurd proteins in neuronal development [8]. However, we were interested to expand on our findings by asking whether other Bacurd family members might also interact with Rnd proteins and play a role in neuronal development. Here, we report that Bacurd1/Kctd13 and Bacurd2/Tnfaip1 are interacting partners to Rnd2 and Rnd3, and our functional studies demonstrate that alterations to *Bacurd* expression disrupt the development of neurons within the mouse cerebral cortex.

## Results

**Fig. 1:**
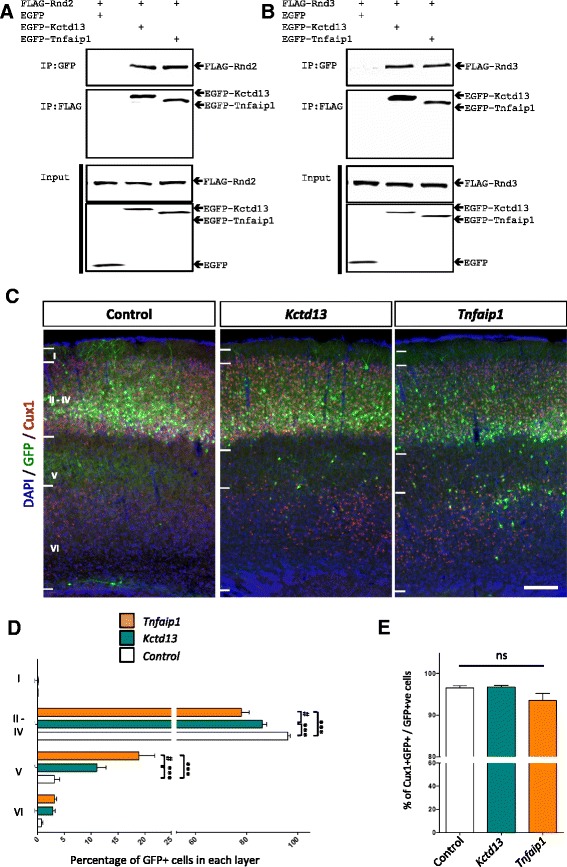
Bacurd1/Kctd13 and Bacurd2/Tnfaip1 are interacting partners to Rnd2/3, and their forced expression impairs the long-term positioning of E14.5-born cortical projection neurons. **a**-**b** Bacurd1/Kctd13 and Bacurd2/Tnfaip1 interact with Rnd2 and Rnd3 in vitro. Immunoprecipitation was performed with cell lysates of HEK293T cells transiently transfected with expression constructs encoding FLAG-tagged Rnd2 together with EGFP, EGFP-Kctd13 and EGFP-Tnfaip1 (**a**), or with FLAG-tagged Rnd3 together with EGFP, EGFP-Kctd13 and EGFP-Tnfaip1 (**b**). Antibodies against EGFP was incubated with the respective lysates, followed by immuno-blotting with antibodies against FLAG-tagged Rnd proteins. A reciprocal experiment was performed in which immunoprecipitation was performed using FLAG antibodies followed by immunoblotting for EGFP. Input panels show Western blot analysis of inputs confirming the presence of all proteins evaluated in this experiment. **c** Forced expression of either Kctd13 or Tnfaip1 results in a significant disruption in the long-term positioning of cortical neurons. Representative images of postnatal day 17 (P17) cortices electroporated with control (GFP only) vector, *Kctd13* or *Tnfaip1* constructs at E14.5 and analysed at P17. **d** There is a significant effect on the distribution of E14.5-labelled cells within the P17 cortex upon forced expression of *Kctd13* or *Tnfaip1* (*N* > 5000 cells from >6 brains per condition; *F*_6,80_ = 24.42; *p* < 0.0001; Two-way ANOVA followed by Bonferroni post-hoc test. Graph plots means ± SEM; *** *p* < 0.001; # *p* < 0.05). **e** Forced expression of Kctd13 or Tnfaip1 does not significantly alter the proportion of GFP-labelled cells which co-express the projection neuron marker Cux1 (*F*_2,20_ = 2.676, *p* = 0.09 One-Way ANOVA, images from at least 6 brains per condition were evaluated). Scale bar represents 100 μm

We performed yeast two-hybrid screens with Rnd2 and Rnd3 bait constructs using a prey cDNA library constructed from embryonic mouse (E15.5) cortex [14]. This led to the cloning of Bacurd1/Kctd13 and Bacurd2/Tnfaip1 as prey interacting partners (Additional file 1: Figure S1A). Complementation assays were performed to confirm specificity of interaction with Rnd2 and Rnd3 (Additional file 1: Figure S1B). We performed reciprocal co-immunoprecipitation experiments with transiently transfected HEKT293T cells and found that EGFP-tagged Bacurd1/Kctd13 and Bacurd2/Tnfaip1 interact with FLAG-tagged Rnd2 and Rnd3, respectively (Fig. 1a, b).

BACURD1/KCTD13 and BACURD2/TNFAIP1 are expressed during mammalian cerebral cortex development in mice and humans (Additional file 1: Figure S1C-D; as well as [8, 15–17]) and we previously reported that forced expression of Bacurd2/Tnfaip1 impaired cell migration within the murine embryonic cortex but not their early neuronal differentiation, as determined by staining for βIII-tubulin [8]. However, it remained unclear as to whether perturbations to *Bacurd* expression levels caused a delay or a defect in the long-term positioning of cortical neurons. Given that elevations in the dosages of *BACURD1*/*KCTD13* and *BACURD2*/*TNFAIP1* genes are associated with structural brain disorders in humans [11, 13], we wanted to study the effects of *Bacurd1/Kctd13* and *Bacurd2/Tnfaip1* overexpression on the development of cortical neurons. For this, we cloned FLAG epitope-tagged expression constructs for Bacurd1/Kctd13 and Bacurd2/Tnfaip1 into a mammalian expression construct which also comprises a GFP cassette (pCIG2) and investigated the consequences of their forced expression within cells of the embryonic E14.5 dorsal telencephalon by *in utero* electroporation. We collected the brains of successfully electroporated postnatal day 17 (P17) mice, a timepoint in which these E14.5-born cortical neurons have completed their migration [1, 18]. As shown in Fig. 1c, forced expression of Bacurd1/Kctd13 or Bacurd2/Tnfaip1 led to a significant impairment in the positioning of GFP-labelled cortical neurons compared with control (GFP only) treatment, observed as a significant decrease in the proportion of GFP+ cells within layers II/III and a concomitant increase in the proportion of layer V cells (Fig. 1d). The relative expression of exogenously derived FLAG-tagged Kctd13 and Tnfaip1 proteins was not significantly different between both treatments (Additional file 2: Figure S2A-B). Also, we found that treatment with *Bacurd1/Kctd13* or *Bacurd2/Tnfaip1* did not significantly alter the neuronal identity of GFP-labelled cells, as determined by co-localisation of the projection neuron marker Cux1 (Fig. 1e and Additional file 2: Figure S2C). Consistent with the notion that appropriate levels of *Bacurd1/Kctd13* and *Bacurd2/Tnfaip1* underpin cortical neuron positioning, we find that knockdown with targeting shRNAs also leads to a disruption in cell positioning (Additional file 2: Figure S2D-H).

**Fig. 2:**
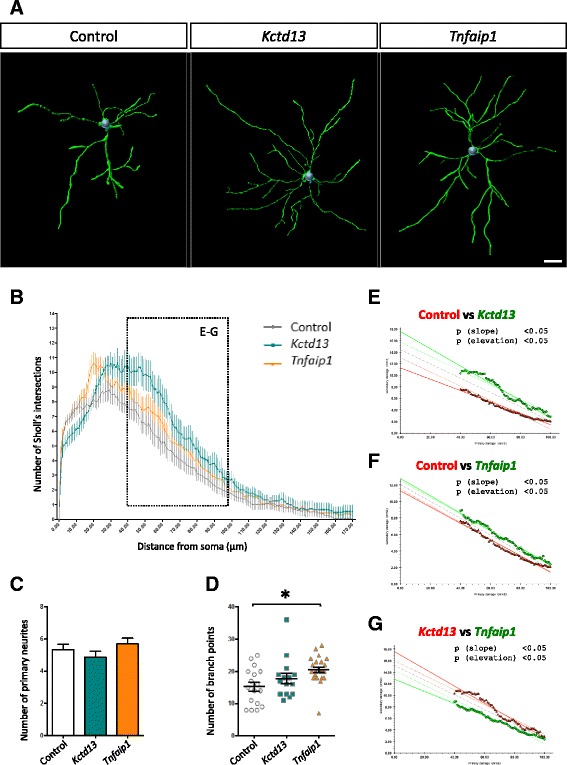
Forced expression of *Kct13* or *Tnfaip1* leads to altered dendritic complexity of layer II/III projection neurons within the P17 mouse cortex. **a** Representative 3D reconstructions of layer II/III control neurons within the P17 mouse cerebral cortex following control, *Kctd13* and *Tnfaip1* treatment at E14.5 (**b**) Sholl analysis reveals significant differences in the dendritic arborisation of neurons upon forced expression of *Kctd13* or *Tnfaip1* (15 and 23 cells analysed respectively) compared with controls (17 cells analysed). **c** The number of primary neurites was not significantly different between control and Kctd13 or Tnfaip1 treatments (*N* > 15 cells from >6 brains per condition; *F*_2,52_ = 1.283; *P* = 0.2858 One way ANOVA followed by Bonferroni post-hoc test). **d** There was a significant increase in the number of branch points upon overexpression of Tnfaip1 when compared with control treatment. (*F*_2,52_ = 4.781; *P* = 0.0124 One way ANOVA followed by Bonferroni post-hoc test). **e**-**g** An Analysis of Covariance (ANCOVA) of Sholl profiles for control and *Kctd13*- or *Tnfaip1*-treated neurons (40–100 μm distal from the soma) reveals significant differences in both the slope (*p* < 0.05) and elevation (*p* < 0.05) of the lines of best fit when comparing with control, indicating that *Kctd13-* and *Tnfaip1-*treated neurons are more complex (**e**-**f**). Also, ANCOVA analysis reveals that *Kctd13-*treated neurons are significantly more complex than *Tnfaip1*-treated neurons (**g**). Scale bar represents 20 μm

To determine whether Bacurd1/Kctd13 and Bacurd2/Tnfaip1 might be important for the terminal differentiation of cortical projection neurons, we analysed GFP-labelled neurons within layers II/III of the cortex by high-power microscopy (see Methods and [19]) and then carried out Sholl analysis, a measure of dendritic complexity [20]. Compared with control, we found that treatment with *Bacurd1*/*Kctd13* or *Bacurd2*/*Tnfaip1* led to a significant enhancement in dendritic complexity (Fig. 2a-b and Additional file 3: Figure S3A-B). While this enhancement was not attributable to changes in the numbers of primary neurites (Fig. 2c), there was a significant difference in dendritic branching, observed as an increase in the number of branch points on *Bacurd2*/*Tnfaip1*-overexpressing cells (Fig. 2d, *F*_2,52_ = 4.781; *P* = 0.0124 One-way ANOVA). There was a modest increase in the number of branch points on *Bacurd1*/*Kctd13*-treated cells which was not significant. In addition, we found that the proximal (40–100 μm) dendritic arbor of *Bacurd1*/*Kctd13-* and *Bacurd2*/*Tnfaip1*-treated neurons exhibited a significant difference in complexity compared with control treatment (Fig. 2b, e-f). Furthermore, the dendritic profiles between *Bacurd1*/*Kctd13-* and *Bacurd2*/*Tnfaip1*-treated neurons were also different (Fig. 2g). Thus, forced expression of *Bacurd1*/*Kctd13* and *Bacurd2*/*Tnfaip1* alters the long-term positioning of neurons and their dendritic maturation, but not their neuronal identity.

**Fig. 3:**
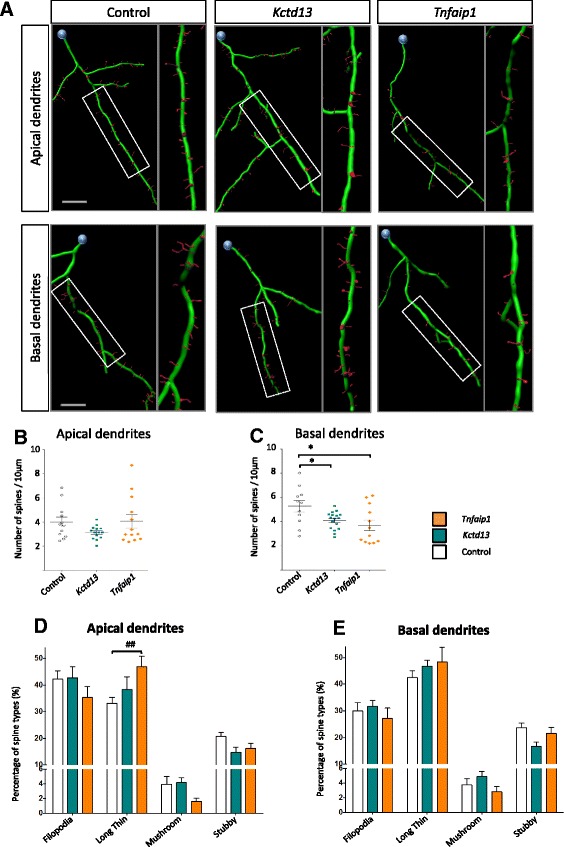
Forced expression of *Kctd13* or *Tnfaip1* leads to altered profiles of dendritic spines of layer II/III cortical neurons within the P17 mouse cortex. **a** Representative 3D reconstructions of the apical and basal dendrites of layer II/III neurons. **b**-**c** Compared with control condition, forced expression of *Kctd13* or *Tnfaip1* did not significantly affect the density on spines on apical dendrites (*F*_2,36_ = 1.82; *P* = 0.1762 One-way ANOVA followed by Bonferroni post-hoc test). In contrast, there was a significant decrease in the density of spines on basal dendrites (*F*_2,37_ = 4.87; *P* = 0.0132 One-way ANOVA followed by Bonferroni post-hoc test). **d**-**e** There was a significant interaction between overexpression of *Kctd13* or *Tnfaip1* and the proportions of dendritic spine morphologies on apical dendrites (*F*_6,136_ = 2.941; *p* < 0.001 Two-way ANOVA followed by Bonferroni post-hoc test) (**d**), but not basal dendrites (*F*_6,148_ = 1.334; *P* = 0.2457 Two-way ANOVA followed by Bonferroni post-hoc test) (**e**). Graph plots mean + SEM; * *p* < 0.05, * *p* < 0.005 compared to control; # *p* < 0.05, ## *p* < 0.005, ### *p* < 0.001 compared with *Kctd13* and *Tnfaip1*. (>10 apical or basal dendrites analysed for each condition). Scale bar represents 10 μm

We analysed the dendritic spine characteristics of *Bacurd*-overexpressing neurons, paying attention to spine densities and spine morphologies along apical and basal dendrites, respectively, using recently reported methods [19]. In apical dendrites, we found that overexpression of *Bacurd1*/*Kctd13* and *Bacurd2*/*Tnfaip1* did not significantly alter the densities of spines (*n* = 10–15 neurons per condition; Fig. 3a-b), but there was a significant interaction between treatment groups and dendritic spine morphologies (Fig. 3d, *F*_6,136_ = 2.941; *P* < 0.001 Two-Way ANOVA), with a notable increase in the proportion of long-thin spines in *Bacurd2*/*Tnfaip1*-overexpressing neurons. In basal dendrites, we found that overexpression of Bacurd1/Kctd13 or Bacurd2/Tnfaip1 resulted in a significant reduction in dendritic spine densities (Fig. 3c), but there was no significant effect on spine morphologies (*P* = 0.257). Thus, overexpression of *Bacurds* leads to distinct effects on the development of cortical neurons in vivo.

## Discussion

This study identifies Bacurd1/Kctd13 and Bacurd2/Tnfaip1 as interacting partners to Rnd2 and Rnd3. We have explored the functions for Bacurd1/Kctd13 and Bacurd2/Tnfaip1 in the development of E14.5-born cortical projection neurons within the postnatal cerebral cortex. Our study suggests that appropriate levels of Bacurds are necessary for the long-term positioning of cortical projection neurons, and we extend from our previous work [8] to show that forced expression of either Bacurd1/Kctd13 or Bacurd2/Tnfaip1 leads to a defect in the radial positioning of cortical projection neurons, rather than a delay in their migration. In addition, we find that treatment with Bacurd1/Kctd13 or Bacurd2/Tnfaip1 leads to distinct effects on the dendritic arborisation of layer II/III cortical projection neurons. Notably, forced expression of Bacurd2/Tnfaip1 results in a more severe impairment in cell positioning as well as neurite branching compared with Bacurd1/Kctd13, and this suggests that Bacurd family members might have distinct functions in neurons. While our in vivo evaluation of the expression constructs for Bacurd1/Kctd13 and Bacurd2/Tnfaip1 indicated that the steady-state levels of exogenously derived protein were not significantly different within cells of the P17 cortex (Additional file 2: Figure S2A-B), we cannot rule out the possibility that different phenotypes in our gain-of-function studies might reflect subtly different levels of overexpression rather than different intrinsic activities in cortical neurons. Nevertheless, our results are consistent with previous findings by Chen and coworkers which demonstrated that Xenopus xBACURDα and xBACURDβ have non-identical functions during gastrulation [21].

During nervous system development, intracellular RhoA signalling in immature neurons is critical for multiple aspects of their maturation, including directional cell migration, neurite and axon outgrowth, dendritic branching and synaptogenesis [22–24]. It is noteworthy that both the Bacurds and the Rnds are important mediators of RhoA signalling, albeit in different ways. The Bacurds recruit Cul3 to ubiquitinate RhoA so as to suppress protein levels, leading to F-actin cytosketal remodelling and inhibition of cell migration [21, 25]. Significantly, a recent study implicated a KCTD13-Cul3-RhoA signalling pathway to be important for human nervous system development and psychiatric disease [16]. In the case of Rnd proteins, both Rnd2 and −3 negatively regulate RhoA signalling to induce cytoskeletal changes in neurons, thereby affecting neurite outgrowth and cell migration in a mechanism involving several protein partners including p190RhoGAP and Plexin B2 [4–6]. Since Rnd2 and Rnd3 regulate multiple steps of radial migration by embryonic cortical neurons as they reach the cortical plate [4, 5], we draw the link here to suggest that such roles for Rnd proteins might also involve functional interactions with Bacurd proteins [8]. In particular, we postulate that Bacurds and Rnds cooperate to influence RhoA signalling in immature neurons for their proper development within the developing cerebral cortex, including their bipolar transition within the IZ and locomotion within the CP as they complete their migration before undergoing dendritic maturation. It remains to be determined if disruptions to Bacurd levels alter the binding of Rnd2 and Rnd3 to their other partners in neurons (such as p190RhoGAP and Plexin B2), or influence RhoA signalling, or both.

We find that overexpression of *Bacurd1*/*Kctd13* or *Bacurd2*/*Tnfaip1* leads to aberrant dendrite arborization, as well as impairments in basal but not apical dendritic spines. It will be important in future studies to determine if down-regulation of endogenous levels of *Bacurds* influence dendritic spines. Notably, our results suggest that Bacurds influence the dendritic maturation of cortical projection neurons in different ways. While this could be explained by differences in developmental timing for apical versus basal dendrites, the mechanistic effects of Bacurd overexpression on dendritic spine properties could also be explained by disruptions to RhoA signalling, a critical mechanism for the formation and stabilization of spines [26]. Altogether, we find that disruptions to endogenous levels of *Bacurd1/Kctd13* and *Bacurd2/Tnfaip1* are likely to impact on the development of neurons, and thus we predict that perturbations to *Bacurd* expression lead to disruptions in neuronal signalling, with ongoing consequences for the balance between neuronal excitation and inhibition within the cerebral cortex. Given the association between elevations in *BACURD1*/*KCTD13* gene dosage and psychosis [11], and *BACURD2/TNFAIP1* with PHACE syndrome (a congenital condition in which infants present with structural brain abnormalities and seizures) [13], it is plausible that excessive *BACURD1/KCTD13* and *BACURD2/TNFAIP1* gene expression may have direct, detrimental effects on the formation of cerebral cortical circuitry, and could lead to neurological dysfunction in humans.

## Methods

### DNA constructs, cell culture, co-immunoprecipitation and western blotting

Complementary DNAs (cDNAs) encoding FLAG-tagged Rnd2, Rnd3, Bacurd1/Kctd13 and Bacurd2/Tnfaip1 and GFP-tagged Bacurd1/Kctd13 and Bacurd2/Tnfaip1 were cloned by PCR and ligated to the mammalian expression construct vector pCMV using compatible ends. Cloned cDNAs encoding Bacurd1/Kctd13 and Bacurd2/Tnfaip1 harbouring MfeI restriction sites were cloned into the EcoRI site of the mammalian expression vector pCIG2. All constructs were sequence verified and plasmids prepared using a Qiagen midipre PLUS kit (Qiagen, Australia?). HEK293T cells were cultured according to conventional protocols, while co-immunoprecipitation studies and Western blotting were performed as previously described [8]. Antibodies used in this study include mouse anti-IgG (millipore) anti-β-actin (Sigma-Aldrich), anti-FLAG (Sigma-Aldrich), anti-GFAP (millipore), anti-FLAG (Cell Signaling), anti-CDP (Cux1, Santa Cruz); and chicken anti-GFP (Abcam). Preimmunised goat serum (Sigma) was used for immunoprecipitation experiments as a control. Cell nuclei were visualized with DAPI (4′6-Diamidino-2-Phenylindole).

### *In utero* electroporation

All procedures were performed in accordance to guidelines the Animal Ethics Committee at Monash University (MARP-2012-069), and in accordance with Institutional guidelines. *In utero* electroporation was performed on E14.5 embryos of time-mated C57/Black6J mice as previously indicated [8]. Injections were carried out with 1 μg/μl of each constituent plasmid. Successfully electroporated animals were verified by GFP epifluorescence inspection through the skull of P0 pups. At postnatal day P17, transcardial perfusion was performed to preserve the brain tissue with fixative (4 % paraformaldehyde/phosphate-buffered saline), and the tissue processed as previously indicated [19]. Further methods related to electroporation experiments performed with small hairpin RNA (shRNA) plasmid constructs are provided in Additional file 4.

### Histological analysis

GFP-labelled P17 neurons were captured as 3-dimensional confocal images of 40 μm-thick mouse brain sections. For cell morphology analysis, GFP-labelled cortical projection neurons residing within layers II/III of the somatosensory cortex of successfully electroporated P17 brains were captured at 40× magnification. For dendritic spine studies, neurites were imaged at 60× magnification, with 1 μm z-stack step size. In both cases, rendering of neuronal cell morphology as well as the identification of dendritic spines was performed manually, while the classification of spine types was automatically calculated based on pre-defined parameters, as described recently [19]. Raw images were analysed and digitally reconstructed using Filament Tracer (Imaris 7.6.2, Bitplane) for the detection of neurons, dendritic trees, axons and spines in 3D.

## Additional files

Additional file 1: Figure S1. Kctd13 and Tnfaip1 are putative interacting partners to Rnd2 and Rnd3 which are expressed in mouse and human tissues. (ZIP 322 kb) Additional file 2: Figure S2. The effects of *Bacurd1/Kctd13* and *Bacurd2/Tnfaip1* on cell positioning and cortical identity. (ZIP 2329 kb) Additional file 3: Figure S3. Analysis of dendritic branching in layer II/III projection neurons of the postnatal P17 cortex. (PDF 650 kb) Additional file 4: Supplementary Methods related to Figure S2 [19, 27]. (DOCX 14.2 kb)

## Abbreviations

CP: cortical plate
Bacurd: BTB/POZ-domain containing Adaptor for Cul3-mediated RhoA Degradation
GFP: Green Fluorescent Protein

## References

[1] Wu Q, Liu J, Fang A, Li R, Bai Y, Kriegstein AR (2014). The dynamics of neuronal migration. Adv Exp Med Biol..

[2] Greig LC, Woodworth MB, Galazo MJ, Padmanabhan H, Macklis JD (2013). Molecular logic of neocortical projection neuron specification, development and diversity. Nat Rev Neurosci.

[3] Pacary E, Azzarelli R, Guillemot F (2013). Rnd3 coordinates early steps of cortical neurogenesis through actin-dependent and -independent mechanisms. Nat Commun..

[4] Pacary E, Heng J, Azzarelli R, Riou P, Castro D, Lebel-Potter M (2011). Proneural transcription factors regulate different steps of cortical neuron migration through Rnd-mediated inhibition of RhoA signaling. Neuron.

[5] Azzarelli R, Guillemot F, Pacary E (2015). Function and regulation of Rnd proteins in cortical projection neuron migration. Front Neurosci..

[6] Azzarelli R, Pacary E, Garg R, Garcez P, van den Berg D, Riou P (2014). An antagonistic interaction between PlexinB2 and Rnd3 controls RhoA activity and cortical neuron migration. Nat Commun..

[7] Heng JI, Nguyen L, Castro DS, Zimmer C, Wildner H, Armant O (2008). Neurogenin 2 controls cortical neuron migration through regulation of Rnd2. Nature.

[8] Gladwyn-Ng IE, Li SS, Qu Z, Davis JM, Ngo L, Haas M (2015). Bacurd2 is a novel interacting partner to Rnd2 which controls radial migration within the developing mammalian cerebral cortex. Neural Dev..

[9] Golzio C, Willer J, Talkowski ME, Oh EC, Taniguchi Y, Jacquemont S (2012). KCTD13 is a major driver of mirrored neuroanatomical phenotypes of the 16p11.2 copy number variant. Nature.

[10] Schwenk J, Metz M, Zolles G, Turecek R, Fritzius T, Bildl W (2010). Native GABA(B) receptors are heteromultimers with a family of auxiliary subunits. Nature.

[11] Steinberg S, de Jong S, Mattheisen M, Costas J, Demontis D, Jamain S (2014). Common variant at 16p11.2 conferring risk of psychosis. Mol Psychiatry.

[12] Blumenthal I, Ragavendran A, Erdin S, Klei L, Sugathan A, Guide JR (2014). Transcriptional consequences of 16p11.2 deletion and duplication in mouse cortex and multiplex autism families. Am J Hum Genet.

[13] Siegel DH, Shieh JT, Kwon EK, Baselga E, Blei F, Cordisco M (2013). Copy number variation analysis in 98 individuals with PHACE syndrome. J Invest Dermatol.

[14] Heng JI, Tan SS (2002). Cloning and characterization of GRIPE, a novel interacting partner of the transcription factor E12 in developing mouse forebrain. J Biol Chem.

[15] Consortium F (2014). the RP, Clst, Forrest AR, Kawaji H, Rehli M et al. A promoter-level mammalian expression atlas. Nature.

[16] Lin GN, Corominas R, Lemmens I, Yang X, Tavernier J, Hill DE (2015). Spatiotemporal 16p11.2 protein network implicates cortical late mid-fetal brain development and KCTD13-Cul3-RhoA pathway in psychiatric diseases. Neuron.

[17] Visel A, Thaller C, Eichele G (2004). GenePaint.org: an atlas of gene expression patterns in the mouse embryo. Nucleic Acids Res.

[18] Kriegstein AR, Noctor SC (2004). Patterns of neuronal migration in the embryonic cortex. Trends Neurosci.

[19] Ngo L, Haas M, Qu Z, Li SS, Zenker J, Teng KS (2014). TUBB5 and its disease-associated mutations influence the terminal differentiation and dendritic spine densities of cerebral cortical neurons. Hum Mol Genet.

[20] Gutierrez H, Dolcet X, Tolcos M, Davies A (2004). HGF regulates the development of cortical pyramidal dendrites. Development.

[21] Chen Y, Yang Z, Meng M, Zhao Y, Dong N, Yan H (2009). Cullin mediates degradation of RhoA through evolutionarily conserved BTB adaptors to control actin cytoskeleton structure and cell movement. Mol Cell.

[22] Govek EE, Newey SE, Van Aelst L (2005). The role of the Rho GTPases in neuronal development. Genes Dev.

[23] Li Z, Van Aelst L, Cline HT (2000). Rho GTPases regulate distinct aspects of dendritic arbor growth in Xenopus central neurons in vivo. Nat Neurosci.

[24] Threadgill R, Bobb K, Ghosh A (1997). Regulation of dendritic growth and remodeling by Rho, Rac, and Cdc42. Neuron.

[25] Sailland J, Tribollet V, Forcet C, Billon C, Barenton B, Carnesecchi J (2014). Estrogen-related receptor alpha decreases RHOA stability to induce orientated cell migration. Proc Natl Acad Sci U S A.

[26] Koleske AJ (2013). Molecular mechanisms of dendrite stability. Nat Rev Neurosci.

[27] Heng JI, Qu Z, Ohtaka-Maruyama C, Okado H, Kasai M, Castro D (2013). The zinc finger transcription factor RP58 negatively regulates Rnd2 for the control of neuronal migration during cortical development. Cereb Cortex.

