# Supplementary material for: Bacurd1/Kctd13 and Bacurd2/Tnfaip1 are interacting partners to Rnd proteins which influence the long-term positioning and dendritic maturation of cerebral cortical neurons
Source: Neural Dev. 2016 Mar 11;11:7. doi: 10.1186/s13064-016-0062-1 (PMC4788816; doi:10.1186/s13064-016-0062-1)
Supplement: Additional file 1: Figure S1. — Kctd13 and Tnfaip1 are putative interacting partners to Rnd2 and Rnd3 which are expressed in mouse and human tissues. (ZIP 322 kb) [file 13064_2016_62_MOESM1_ESM.zip › legends.docx]

Kctd13 and Tnfaip1 are putative interacting partners to Rnd2 and Rnd3 which are expressed in mouse and human tissues. (A) A yeast two-hybrid interaction screen was performed with an E15.5 embryonic cortex library, using truncated versions of Rnd2 and Rnd3 as baits, each lacking their C-terminal (CAAX) motifs.

Growth in selection media (lacking histidine and adenine) is indicated by ‘+++’, while lack of growth is represented with a “-”. (B) Growth of yeast co-transfected with various combinations of bait and prey plasmids, as indicated. Cell growth in nutritional selection media is observed when cells are co-transfected with Kctd13 prey and Rnd baits, but not p53 and pLaminC baits. Cell growth is observed when cells are cotransfected with Tnfaip1 prey and Rnd baits, but not p53 and pLaminC baits. Positive control (p53 bait and pSV40 prey) and negative control interactions (pLaminC bait and pSV40 prey) are also represented. (C) Images of in situ hybridisation signals for Rnd2, Rnd3, Bacurd1/Kctd13 and Bacurd2/Tnfaip1 on sagittal sections of E14.5 mouse embryos from the expression atlas GenePaint, with details of cRNA probes and NBT/BCIP colorimetric detection as reported [17]. Asterisk denotes the dorsal telencephalon within which prominent Rnd2 and Rnd3 expression is evident, while signals for Bacurd1/Kctd13 and Bacurd2/Tnfaip1 appear more diffuse. (D) A survey of RND2, RND3, BACURD1/KCTD13 andBACURD2/TNFAIP1 mRNA expression in human brain tissue from human fetal (pink bars) and adult (yellow bars) samples using Capped Analysis of Gene Expression (CAGE) in FANTOM5 [15]. Quantitative data was normalized across libraries and expressed as Tags Per Million (TPM) mapped reads in a given CAGE library. Where multiple samples were available from a given tissue, data is plotted as an mean ± standard deviation. As a guide, expression levels at 10 TPM reflect approximately 3 copies of a given transcript within a cell.
