# Supplementary material for: Bacurd1/Kctd13 and Bacurd2/Tnfaip1 are interacting partners to Rnd proteins which influence the long-term positioning and dendritic maturation of cerebral cortical neurons
Source: Neural Dev. 2016 Mar 11;11:7. doi: 10.1186/s13064-016-0062-1 (PMC4788816; doi:10.1186/s13064-016-0062-1)

Additional File 1, Figure S1 (part 1)

A

Bait	Prey	Growth in media<br>- *his*, -*ade*
Rnd2$^{1 - 223}$	Kctd13$^{1 - 329}$	+++
Rnd3$^{1 - 240}$	Kctd13$^{1 - 329}$	+++
pLaminC	Kctd13$^{1 - 329}$	-
p53	Kctd13$^{1 - 329}$	-
Rnd2$^{1 - 223}$	Tnfaip1$^{1 - 316}$	+++
Rnd2$^{1 - 223}$	Tnfaip1$^{242 - 316}$	+++
Rnd3$^{1 - 240}$	Tnfaip1$^{242 - 316}$	+++
pLaminC	Tnfaip1$^{242 - 316}$	-
p53	Tnfaip1$^{242 - 316}$	-
Rnd2$^{1 - 223}$	SV40	-
Rnd3$^{1 - 240}$	SV40	-
p53	SV40	+++
pLaminC	SV40	-

B

Rnd2$^{1-223}$ bait | Rnd3$^{1-240}$ bait | pLaminC bait | p53 bait

Tnfaip1$^{1-316}$ prey

Kctd13$^{1-329}$ prey

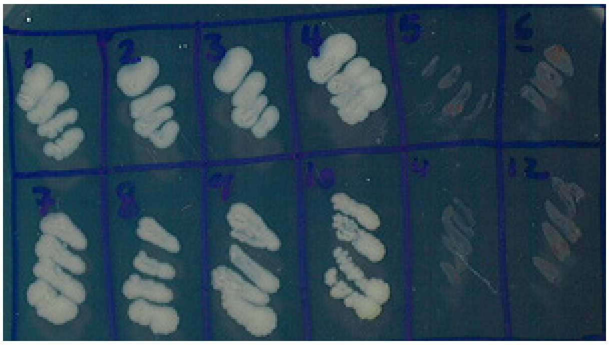

p53 bait | pLaminC bait | Rnd2$^{1-223}$ bait | Rnd3$^{1-240}$ bait

pSV40 prey

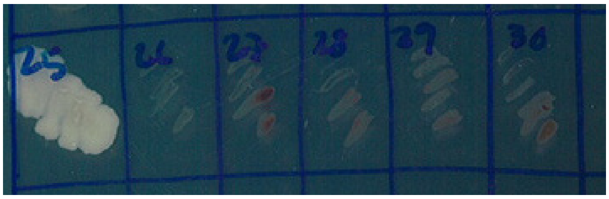

Supplement: Additional file 1: Figure S1. — Kctd13 and Tnfaip1 are putative interacting partners to Rnd2 and Rnd3 which are expressed in mouse and human tissues. (ZIP 322 kb) [file 13064_2016_62_MOESM1_ESM.zip › NDEV-D-15-00024_Additional File 1 Figure S1A_B.pdf]
