# Supplementary figures and images for: Bacurd1/Kctd13 and Bacurd2/Tnfaip1 are interacting partners to Rnd proteins which influence the long-term positioning and dendritic maturation of cerebral cortical neurons
Source: Neural Dev. 2016 Mar 11;11:7. doi: 10.1186/s13064-016-0062-1 (PMC4788816; doi:10.1186/s13064-016-0062-1)

C

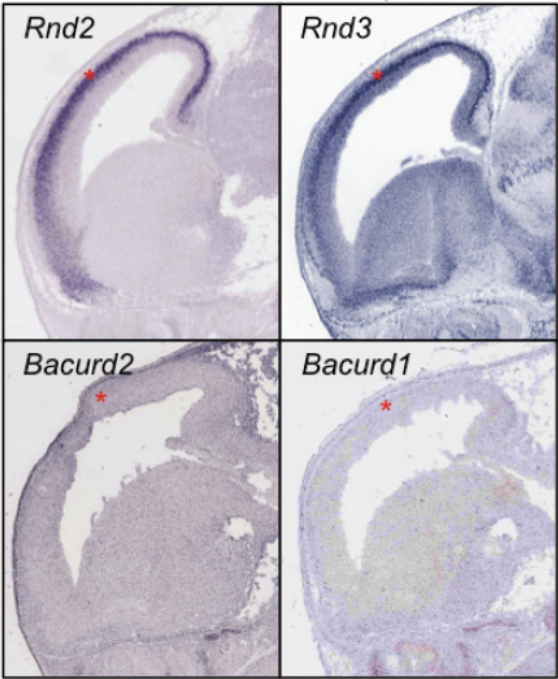

D

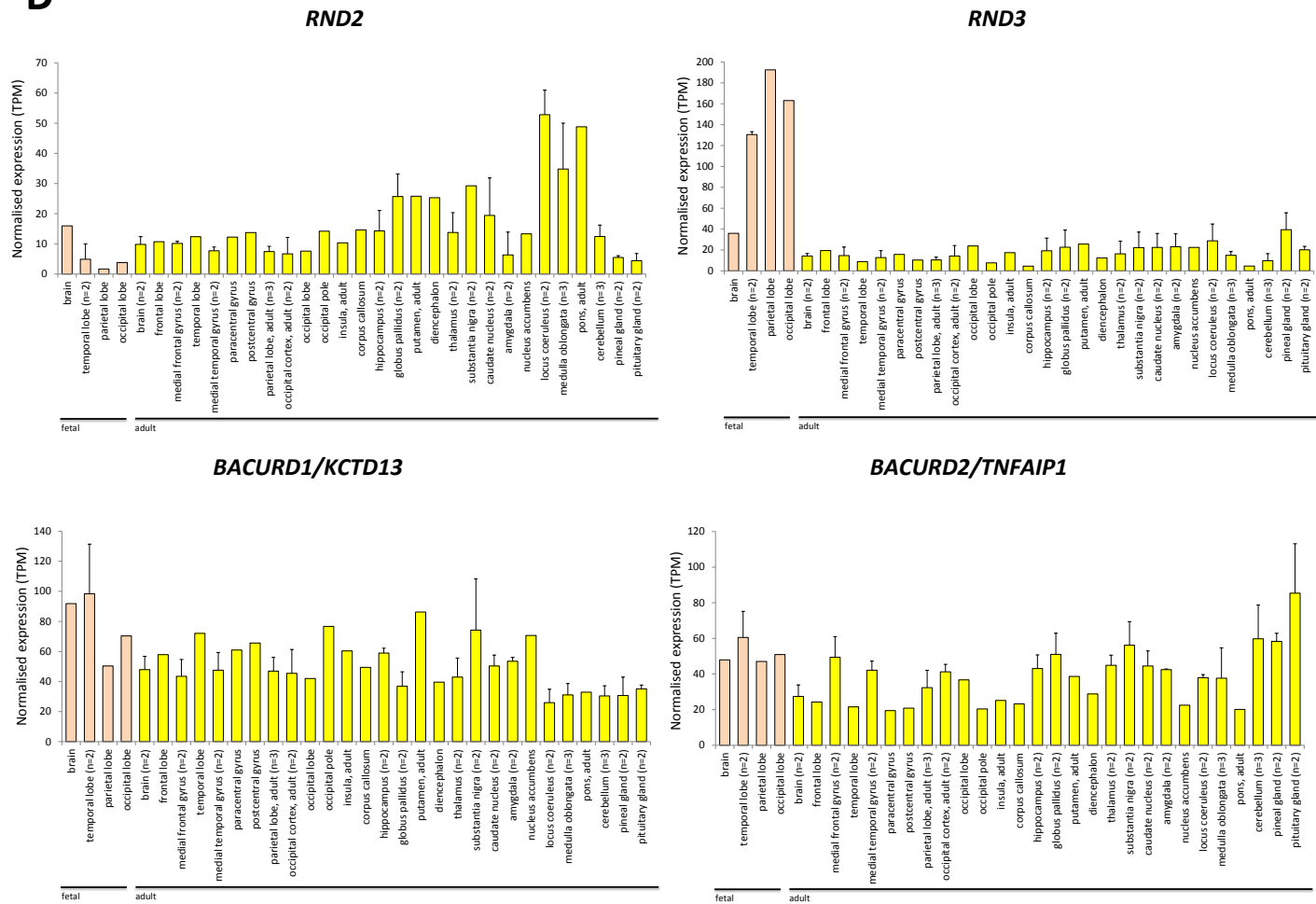

Supplement: Additional file 1: Figure S1. — Kctd13 and Tnfaip1 are putative interacting partners to Rnd2 and Rnd3 which are expressed in mouse and human tissues. (ZIP 322 kb) [file 13064_2016_62_MOESM1_ESM.zip › NDEV-D-15-00024_Additional File 1 Figure S1C_D.pdf]

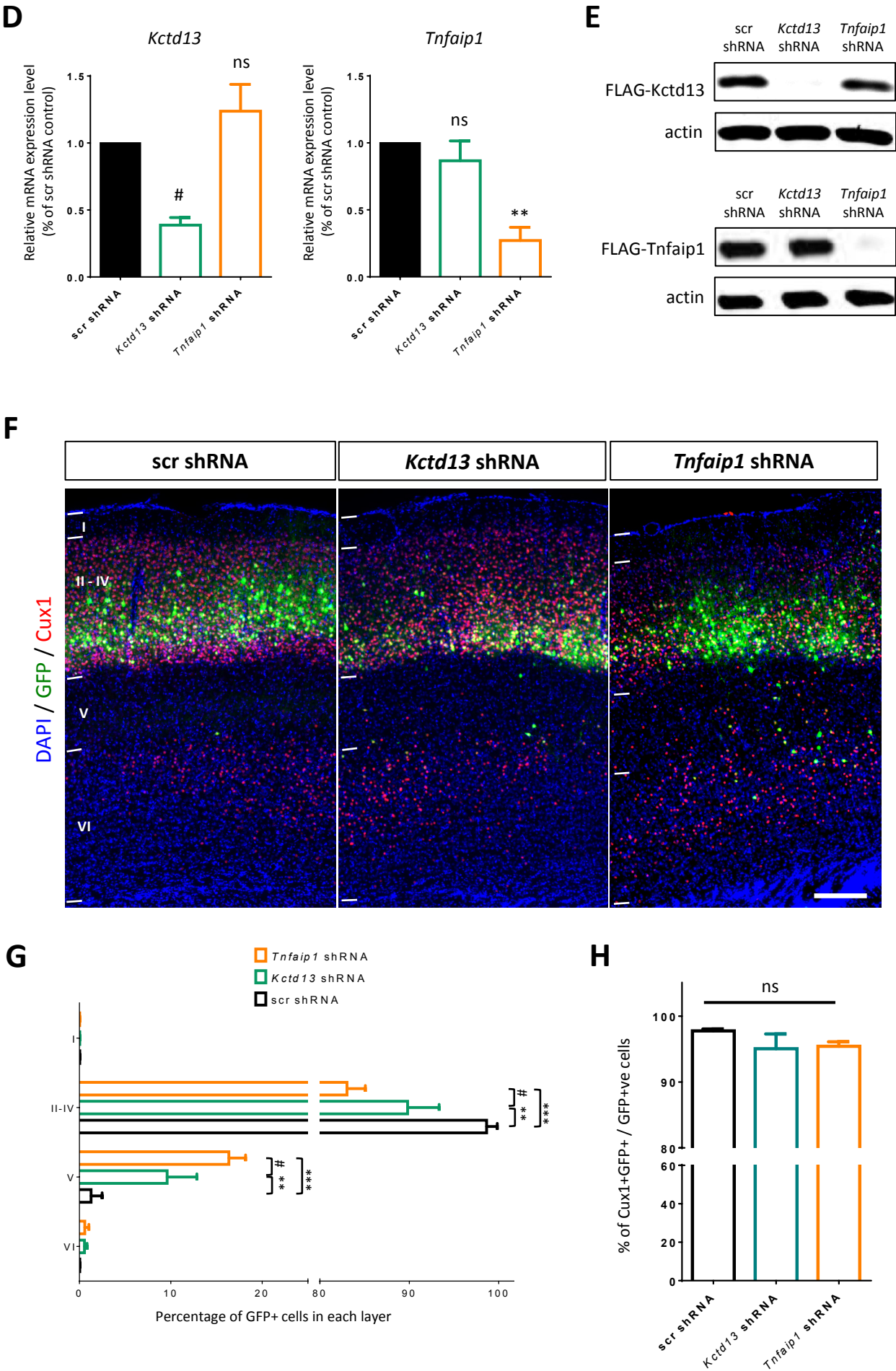

Supplement: Additional file 2: Figure S2. — The effects of Bacurd1/Kctd13 and Bacurd2/Tnfaip1 on cell positioning and cortical identity. (ZIP 2329 kb) [file 13064_2016_62_MOESM2_ESM.zip › NDEV-D-15-00024_Additional File 2 Figure S2D_H.pdf]

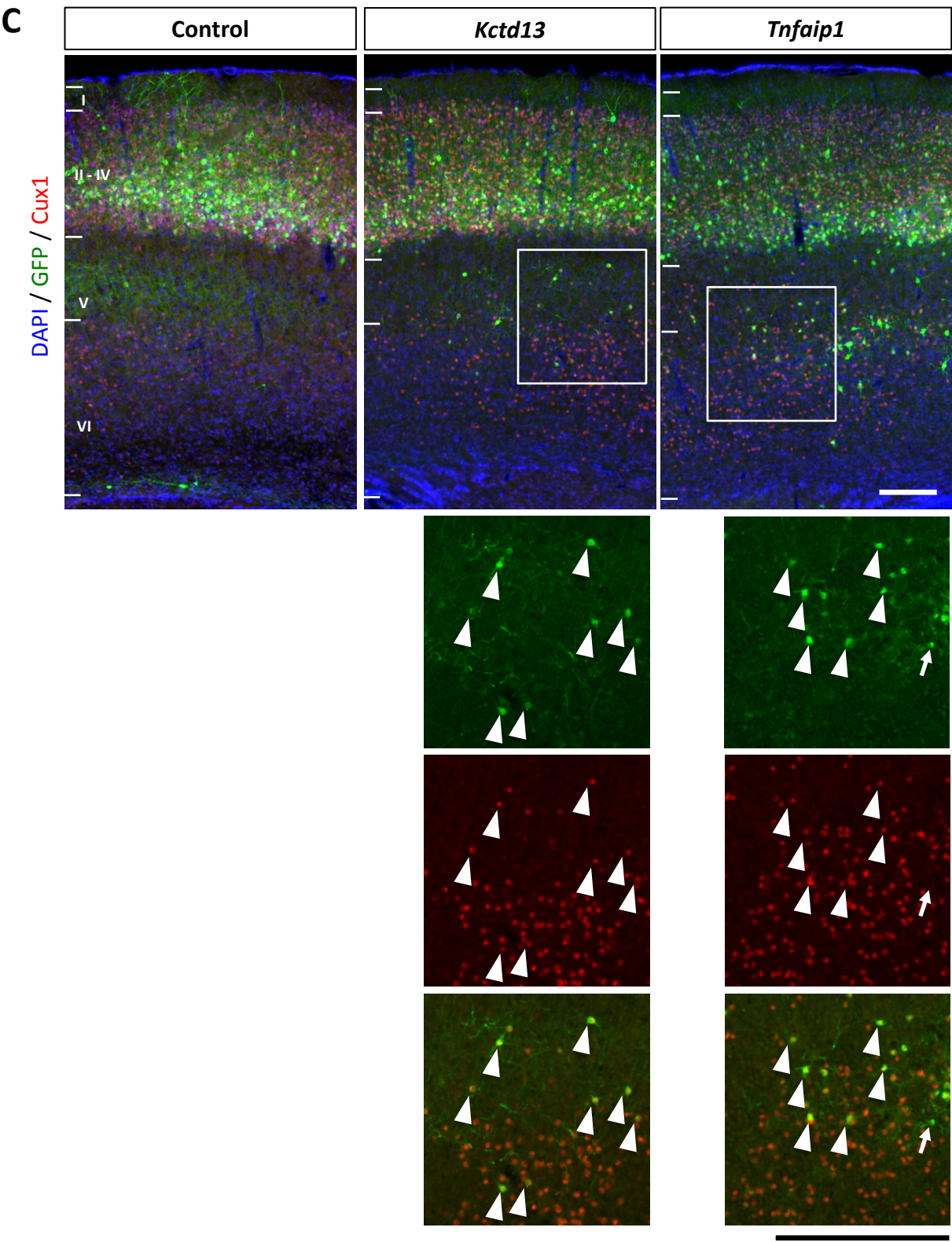

Supplement: Additional file 2: Figure S2. — The effects of Bacurd1/Kctd13 and Bacurd2/Tnfaip1 on cell positioning and cortical identity. (ZIP 2329 kb) [file 13064_2016_62_MOESM2_ESM.zip › NDEV-D-15-00024_Additional File 2 Figure S2C.pdf]

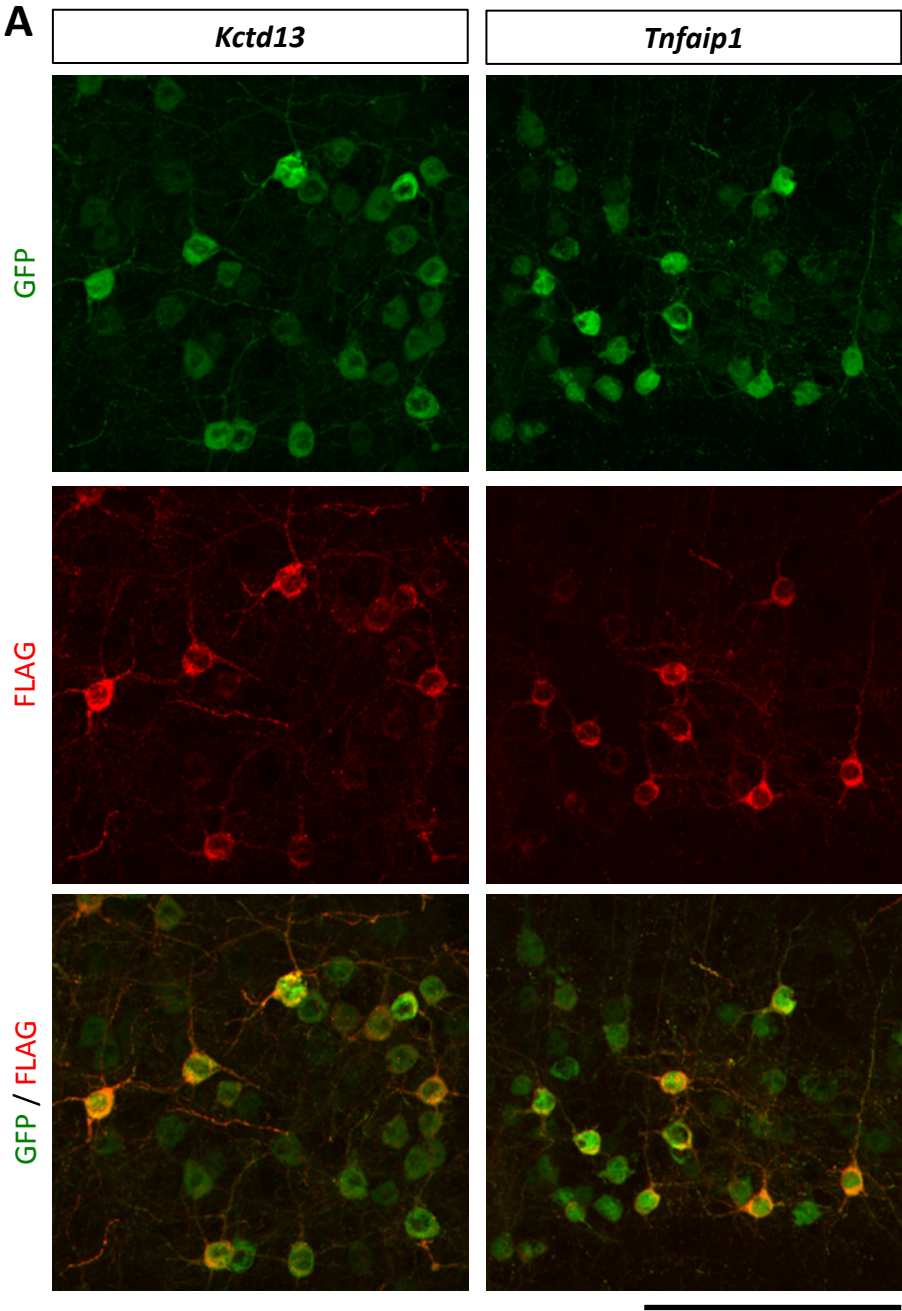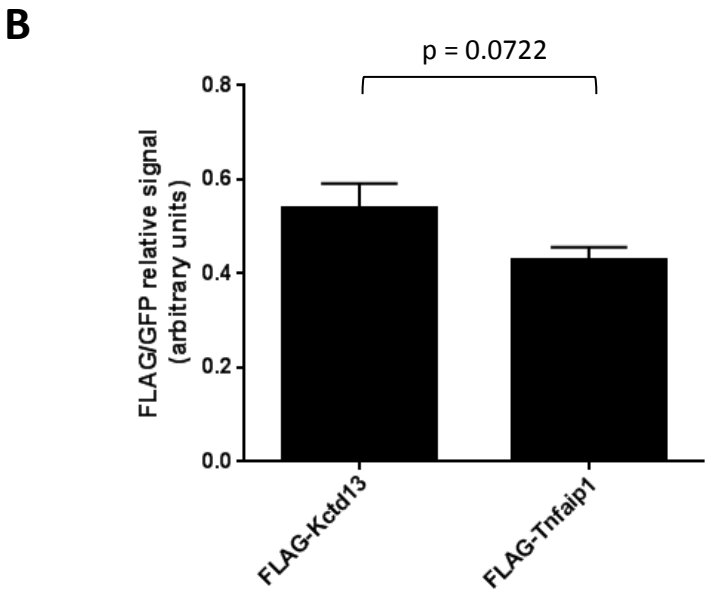

Supplement: Additional file 2: Figure S2. — The effects of Bacurd1/Kctd13 and Bacurd2/Tnfaip1 on cell positioning and cortical identity. (ZIP 2329 kb) [file 13064_2016_62_MOESM2_ESM.zip › NDEV-D-15-00024_Additional File 2 Figure S2A_B.pdf]

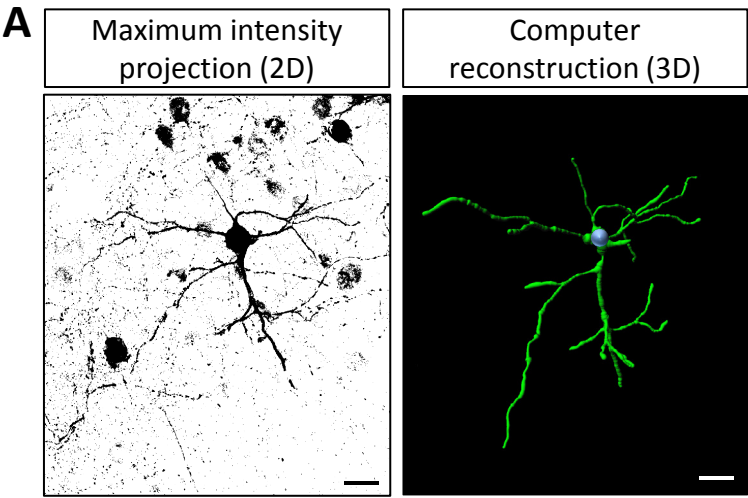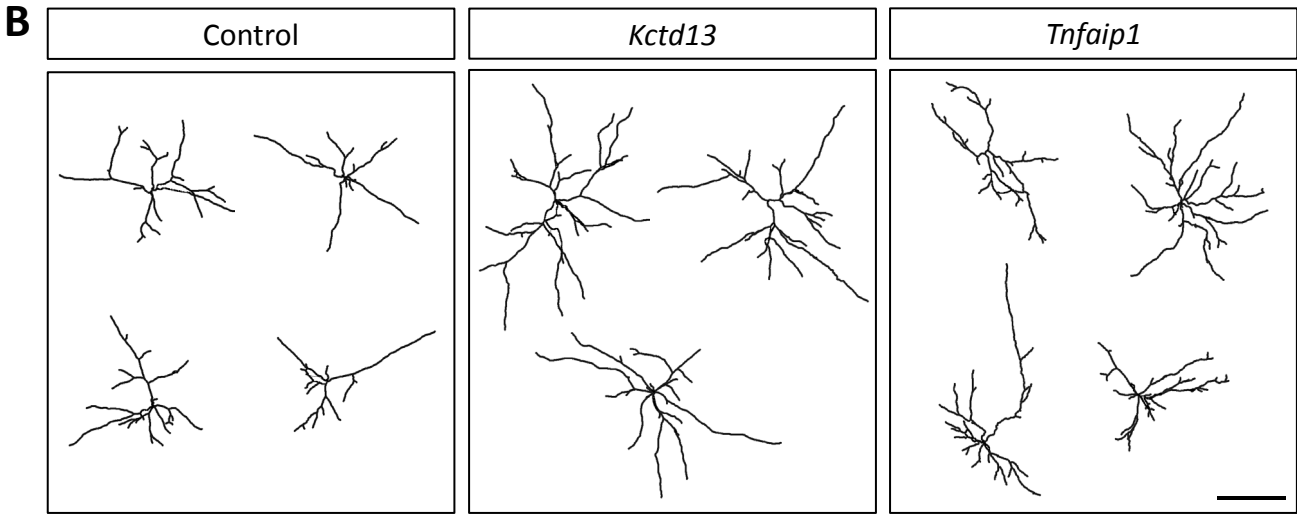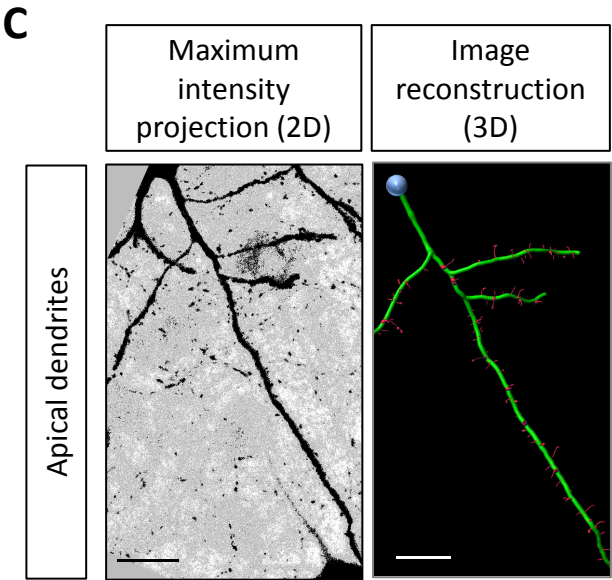

Supplement: Additional file 3: Figure S3. — Analysis of dendritic branching in layer II/III projection neurons of the postnatal P17 cortex. (PDF 650 kb) [file 13064_2016_62_MOESM3_ESM.zip › 13064_2016_62_MOESM3_ESM.pdf]
