# Supplementary material for: Bacurd1/Kctd13 and Bacurd2/Tnfaip1 are interacting partners to Rnd proteins which influence the long-term positioning and dendritic maturation of cerebral cortical neurons
Source: Neural Dev. 2016 Mar 11;11:7. doi: 10.1186/s13064-016-0062-1 (PMC4788816; doi:10.1186/s13064-016-0062-1)
Supplement: Additional file 2: Figure S2. — The effects of Bacurd1/Kctd13 and Bacurd2/Tnfaip1 on cell positioning and cortical identity. (ZIP 2329 kb) [file 13064_2016_62_MOESM2_ESM.zip › legends.docx]

The effects of Bacurd1/Kctd13 and Bacurd2/Tnfaip1 on cell positioning and cortical identity. (A-B) Cortical cells within the P17 cerebral cortex were immunostained for GFP and FLAG following in utero electroporation with pCIG-FLAG-Bacurd1/Kctd13 and pCIG-FLAG-Bacurd2/Tnfaip1, respectively.

Quantification reveals that the immunofluorescence signal for FLAG relative to GFP was not significantly different between Kctd13 and Tnfaip1 treatments (P = 0.0722, unpaired student’s t-test, >35 cells counted per condition). (C) Forced expression of Kctd13 or Tnfaip1 does not significantly alter the expression of the

projection neuron marker Cux1 in GFP-electroporated cells. Magnified images of boxed regions show GFPlabelled cells within layers V and VI which co-express Cux1 (in red), and identified with a white arrowhead. A GFP+/Cux1- cell is indicated with a white arrow in the case ofTnfaip1-overexpression. There was no

significant difference in the proportion of GFP+/Cux1+ cells upon Tnfaip1 overexpression. (D) Verification of Bacurd1/Kctd13 shRNA and Bacurd2/Tnfaip1 shRNA constructs for specific knockdown of endogenous targets through transient transfections of Neuro2a cells followed by Q-RT-PCR analysis. A previously characterised non-targeting scrambled shRNA (scr) hairpin was used as a control [27]. As shown, Kctd13 shRNA but not Tnfaip1 shRNA treatment leads to knockdown of endogenous Kctd13 (F2,9 = 13, P = 0.002 One-Way ANOVA), while Tnfaip1 shRNA but not Kctd13 shRNA treatment leads to knockdown of endogenous Tnfaip1 (F2,9 = 14, P = 0.0016 One-Way ANOVA). These selected shRNAs were one of four independent shRNAs which target distinct sequences for Bacurd1/Kctd13 and for Bacurd2/Tnfaip1, respectively (not shown). Bars represent mean ± SEM from biological triplicates. (E) Western blotting analysis of scr (non-targeting), Bacurd1/Kctd13 shRNA and Bacurd2/Tnfaip1 shRNA demonstrates specificity of knockdown of FLAG-tagged Bacurds. Neuro2a cells were co-transfected with shRNA plasmids and expression constructs as indicated, then protein lysates were collected 48 h later for immunoblotting. (F) Suppression of endogenous Kctd13 or Tnfaip1 by RNAi results in a long-term positioning defect of E14.5-born cortical neurons within the P17 cortex. Representative images of postnatal day 17 (P17) cortices

electroporated with non-targeting (scr) shRNA vector, Kctd13 shRNA or Tnfaip1 shRNA constructs, each of which comprise a GFP expression cassette. (G) There is a significant interaction between shRNA treatment and the distribution of GFP-labelled cells within the P17 cortex (at least 5500 cells from 5–7 brains per

condition were counted; F6,60 = 10; P < 0.0001; Two-way ANOVA followed by Bonferroni post-hoc test. Bars represent means ± SEM; *** p < 0.001, ** p < 0.01, # p < 0.05). (H) Suppression of Kctd13 or Tnfaip1 by RNAi does not significantly alter the proportion of GFP-labelled cells which co-express the projection neuron marker Cux1 (F2,16 = 0.66, P = 0.53 One-Way ANOVA, images from 5–7 brains per condition were evaluated). Scale bars in (A), (C) and (E) represent 100 μm.
