# Supplementary material for: Bacurd1/Kctd13 and Bacurd2/Tnfaip1 are interacting partners to Rnd proteins which influence the long-term positioning and dendritic maturation of cerebral cortical neurons
Source: Neural Dev. 2016 Mar 11;11:7. doi: 10.1186/s13064-016-0062-1 (PMC4788816; doi:10.1186/s13064-016-0062-1)
Supplement: Additional file 3: Figure S3. — Analysis of dendritic branching in layer II/III projection neurons of the postnatal P17 cortex. (PDF 650 kb) [file 13064_2016_62_MOESM3_ESM.zip › legends.docx]

Analysis of dendritic branching in layer II/III projection neurons of the postnatal P17 cortex. (A) Monochromatic image of the Maximum Intensity Projection (2D) for a recorded P17 cortical projection neuron alongside the corresponding 3D model (seeMethods). Confocal image stacks were digitally reconstructed using Imaris Software to build 3D models of branches. (B) Ball-and-stick models of

representative neurons analysed for each treatment (Control, Kctd13 overexpression, Tnfaip1 overexpression). (C) Representative image of a maximum intensity projection (2D) recorded from an apical dendrite captured by confocal microscopy. Scale bar represents 20 μm (A-B) and 10 μm (C) respectively.
