# Supplementary material for: Bacurd1/Kctd13 and Bacurd2/Tnfaip1 are interacting partners to Rnd proteins which influence the long-term positioning and dendritic maturation of cerebral cortical neurons
Source: Neural Dev. 2016 Mar 11;11:7. doi: 10.1186/s13064-016-0062-1 (PMC4788816; doi:10.1186/s13064-016-0062-1)
Supplement: Additional file 4: — Supplementary Methods related to Figure S2 [19, 27]. (DOCX 14.2 kb) [file 13064_2016_62_MOESM4_ESM.docx]

***Supplementary Methods***

Sequences for targeting short hairpin RNA (shRNA) constructs were derived from Dharmacon ON-TARGETplus SMARTpool siRNA: mouse *Bacurd1/Kctd13* (J-055954-07 5’-GUAACAACAAGUACUCU-3’ sense) and *Bacurd2/Tnfaip1* (J-058905-08 5’-GGAAACACTCAATGTCCTA-3’ sense). Hairpin oligonucleotides sequences were cloned into pSilencer expression vector. A previously characterised non-targeting scrambled shRNA (scr) hairpin was used as a control [27]. All plasmids were sequenced verified and prepared using Qiagen DNA (endolow midiprep) purification, and eluted in water. The efficacy of knockdown by shRNAs was evaluated by Lipofectamine™ transfection of shRNAs into neuroblastoma Neuro-2A cells, followed by Quantitative real-time Polymerase Chain Reaction (Q-RT-PCR) 48 h after transfection, as previously reported [19], but explained here in brief. Total mRNA was first extracted from transfected cells (ISOLATE II RNA, BioLine BIO-52073) and then subject to reverse transcription (SuperScript® IV Reverse Transcriptase, Life Technologies 18090010). Q-RT-PCR involved exon spanning primers for *Kctd13* (Forward: 5’- AAAAATTGCCGAGGTGTGCTG, reverse: 5’- AAGATCCGAGCCTCAGGGAAT) and *Tnfaip1* (5’- ACCTCATTCAAGGGCTGGTG, reverse: 5’- CTCAGGGACGTGATGATGGG), and with *Hsp90ab1* (forward: 5’- ACCTGGGAACCATTGCTAAG, reverse: 5’-AGAATCCGACACCAAACTGC) and *Pgk1* (forward: 5’-AAACTCAGCCATGTGAGCACT, reverse: 5’-ACTTAGGAGCACAGGAACCAAA) as housekeeping genes. Each Q-RT-PCR reaction (SensiMix SYBR Hi-ROX, Bioline BIO-52073) comprised 10 ng/μL template cDNA, 5 μM target gene primers or 10 μM housekeeping gene primers. The level of detected *Kctd13* and *Tnfaip1* was expressed relative to *Pgk1.*
